# Effect of Hydroxyapatite Augmentation on Femoral Lag Screw Insertion in Intertrochanteric Femoral Fractures in the Elderly: A Biomechanical Study

**DOI:** 10.7759/cureus.81448

**Published:** 2025-03-30

**Authors:** Yusuke Hattori, Takuya Usami, Gen Kuroyanagi, Hidetoshi Iwata, Isato Sekiya, Waguri-Yuko Nagaya, Hideki Murakami, Naoya Takada

**Affiliations:** 1 Orthopaedic Surgery, Nagoya City University Graduate School of Medical Sciences, Nagoya, JPN; 2 Rehabilitation Medicine, Nagoya City University Graduate School of Medical Sciences, Nagoya, JPN; 3 Orthopaedic Surgery, Aichi Prefectural Welfare Federation of Agricultural Cooperatives Kainan Hospital, Yatomi, JPN; 4 Orthopaedic Surgery, Nagoya City University East Medical Center, Nagoya, JPN

**Keywords:** biomechanical study, bone mineral density, extracted human femoral heads, hydroxyapatite augmentation, intertrochanteric femoral fractures, lag screw fixation strength

## Abstract

Background: This study aimed to investigate the effect of hydroxyapatite (HA) augmentation on the maximum lag screw insertion torque (T_Max_) using extracted human femoral heads.

Materials and methods: Hemispherical femoral heads extracted from patients who underwent bipolar hip arthroplasty were used. These samples were divided into the HA treatment group (HA group) and the non-HA treatment group (N group). After drilling, HA was inserted into the femoral heads in the HA group. A lag screw was inserted into the femoral head using a dedicated jig in both groups. The bone mineral density of the uninjured contralateral femoral neck (f-BMD), T_Max_, and the T_Max_/f-BMD ratio were evaluated.

Results: Twenty-seven samples were analyzed in this study: 14 samples in the HA group and 13 samples in the N group. No significant differences were observed in f-BMD and T_Max_ between the two groups (HA: 0.49 ± 0.11 vs. N: 0.48 ± 0.092 g/cm^2^, p = 0.90; HA: 5.6 ± 1.9 vs. N: 4.3 ± 1.9 N･m, p = 0.78). Considering bone mineral density, the HA group showed a significantly higher T_Max_/f-BMD ratio than the N group (11.7 ± 3.3 vs. 8.9 ± 3.2 N･m･cm^2^/g, p = 0.036). Correlation coefficients between f-BMD and T_Max_in both groups were moderately strong (r = 0.50 vs. 0.50).

Conclusion: Our results suggest that HA augmentation improves lag screw fixation strength in the treatment of osteoporotic intertrochanteric femoral fractures.

## Introduction

Intertrochanteric femoral fractures frequently occur in the elderly and result from low-energy trauma due to bone fragility [1,2]. The mortality rate in the first year after intertrochanteric femoral fractures has been reported to be 10-30% [2,3]. Intramedullary nails are generally used for the surgical treatment of intertrochanteric femoral fractures [2,4]. However, literature has reported poor prognostic factors for intertrochanteric femoral fracture treatment, including lesser trochanteric fractures, reverse oblique fractures, and severe osteoporosis [3,5]. Furthermore, postoperative complications such as femoral head collapse or lag screw cut-out have still been reported with the treatment of intertrochanteric femoral fractures using intramedullary nails. These complications are related to osteoporotic bones [6]. Therefore, further treatment strategies for osteoporotic bones need to be clarified.

Some studies have reported that screw insertion torque correlates with bone mineral density and pullout strength [7-9]. Thus, increasing the screw insertion torque can be a new treatment option for osteoporotic bones. Bone cement augmentation has been reported to improve mechanical fixation strength, rotational torque resistance, and pullout strength [10,11]. However, bone cement augmentation is sometimes at risk of osteonecrosis due to heat and chemical reactions [12,13].

Hydroxyapatite (HA) has been used in numerous orthopedic fields, including spine and trauma [14-16]. HA-coated implants have been reported to reduce the long-term risk of cut-outs in the treatment of osteoporotic intertrochanteric femoral fractures [17]. Additionally, Nakajima et al. reported that a 100% HA cylinder, Neobrace® (Aimedic MMT Co., Ltd., Tokyo, Japan), increased rotational torque for intramedullary nails, although this study was conducted using rigid polyurethane foam to mimic osteoporotic bone [18]. Despite these findings, no biomechanical studies have clarified the efficacy of HA augmentation to the lag screw hole in the treatment of intertrochanteric fractures using extracted human femoral heads. The present study aimed to investigate the effect of HA augmentation on screw insertion torque in extracted human femoral heads. We hypothesized that HA augmentation would enhance lag screw fixation strength in the treatment of osteoporotic intertrochanteric femoral fractures.

This article was previously presented as a meeting abstract at the 25th EFORT Annual Congress.

## Materials and methods

Femoral head preparation

**Figure 1:**
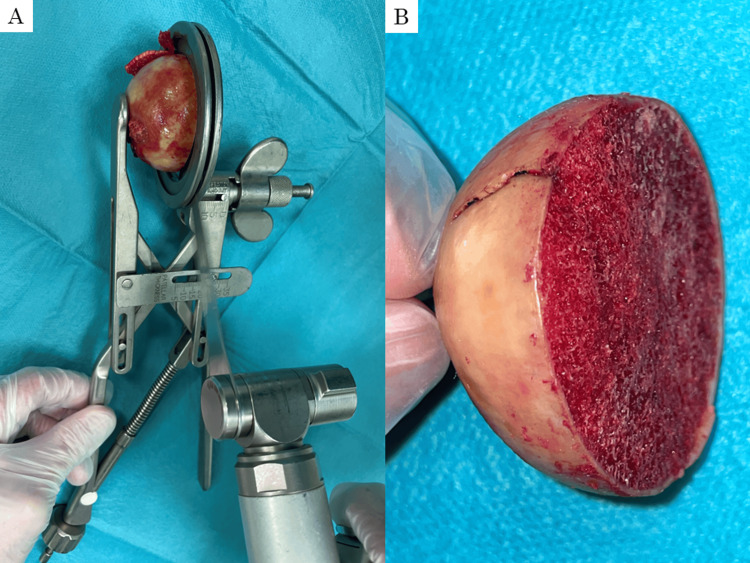
Extracted human femoral head The femoral head was cut into a hemispherical shape using a special cutting jig and a bone saw (A). The thickness of the hemispheres is 35 mm (B).

This study was approved by the Research Ethics Committee of Kainan Hospital (No. 20200221-01). This study included 27 patients who underwent bipolar hip arthroplasty for femoral neck fractures at our institution from February to June 2021. Written informed consent was firmly obtained from all patients before enrollment. Patients with a history of contralateral proximal femoral surgeries and damaged femoral heads were excluded from the study. The femoral heads were nondestructively removed during the surgeries. To mitigate the effect of the femoral neck fractures, the heads were cut into a hemispherical shape using a bone saw at 35 mm from the tip of the femoral heads (Figure 1).

Group randomization

Twenty-seven femoral heads were randomly divided into two groups: the HA treatment group (HA group) with Neobrace® for HA augmentation and the non-HA treatment group (N group) without using Neobrace®. The effect of Neobrace® on mechanical testing was evaluated by comparing the HA and N groups.

Biomechanical testing and data collection

**Figure 2:**
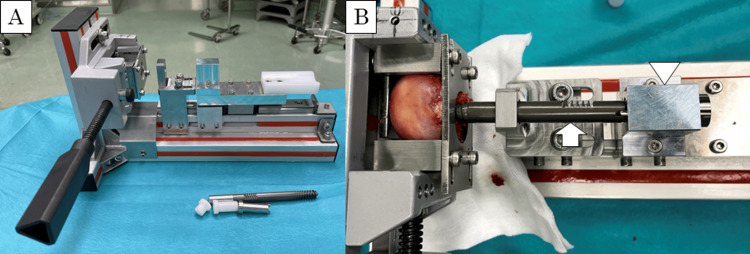
Custom-made testing jig A custom-made testing jig was used. A guide wire and a screw were inserted coaxially along the rail (A). The screw (arrows) was prevented from advancing at 5 mm from the tip of the femoral head by the stopper (arrowheads) (B).

**Figure 3:**
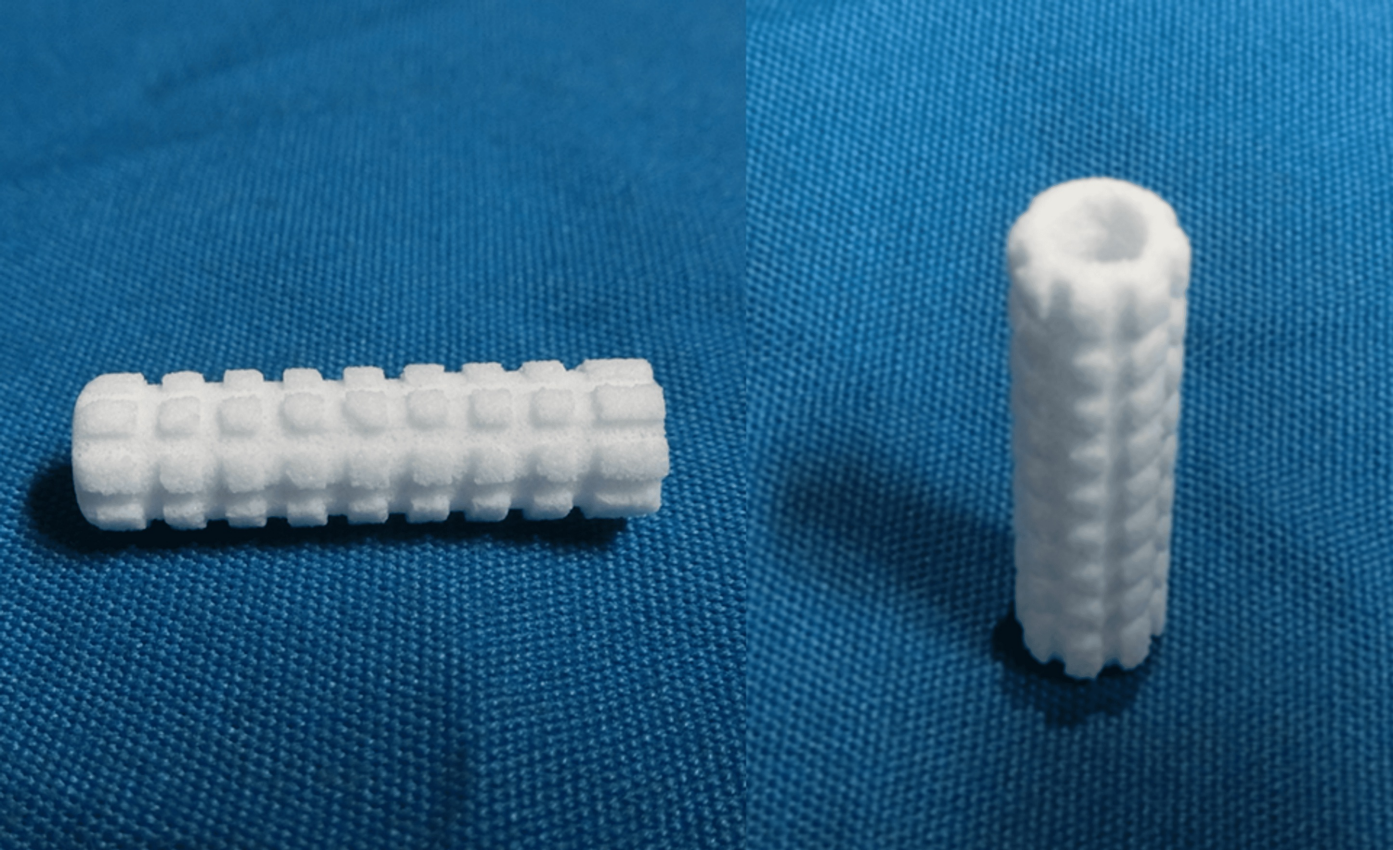
Neobrace® Neobrace® (Aimedic MMT Co., Ltd., Tokyo, Japan) was used for HA augmentation. Neobrace® has a cylindrical shape and can be inserted along a guide wire through the drilled hole. HA, hydroxyapatite

To perform an accurate evaluation, we created a custom-made testing jig to insert the lag screw into the extracted femoral head under controlled conditions to standardize the lag screw insertion position, depth, and soft tissue interference. The jig was designed to insert a guide wire coaxially toward the tip of the femoral head along a rail (Figure 2A). Using a 2.4 mm diameter guide wire, the femoral head was drilled with a 7.6 mm diameter drill bit until the distance between the tip of the femoral head and the drill tip reached 5 mm. In the HA group, one Neobrace® (inner diameter 3.5 mm, outer diameter 7.0 mm, and length 12.5 mm) was inserted into the femoral head along the guide wire through the drilled hole (Figure 3). INTERTAN® lag screw (Smith & Nephew, Memphis, TN, USA) (diameter 11 mm and length 105 mm) was inserted into the femoral heads in both groups. Screw insertion torque was measured using a digital torque gauge (HTGA-10N, Imada Co., Ltd., Tokyo, Japan) and recorded using dedicated software (Force Recorder, Imada Co., Ltd., Tokyo, Japan). The sampling rate was 2000 Hz. The stopper placed behind the lag screw stopped the screw from advancing at 5 mm from the tip of the femoral head, ensuring a consistent tip-apex distance across all samples (Figure 2B). After reaching the controlled distance, the screw continued to turn until it slipped, with the insertion torque progressively increasing. Patients’ operated side, sex, and ages were obtained from the medical chart. The bone mineral density of the uninjured contralateral femoral neck (f-BMD) was measured using dual-energy X-ray absorptiometry systems (DCS-900EX, Hitachi, Ltd., Tokyo, Japan).

Data analysis and calculation

**Figure 4:**
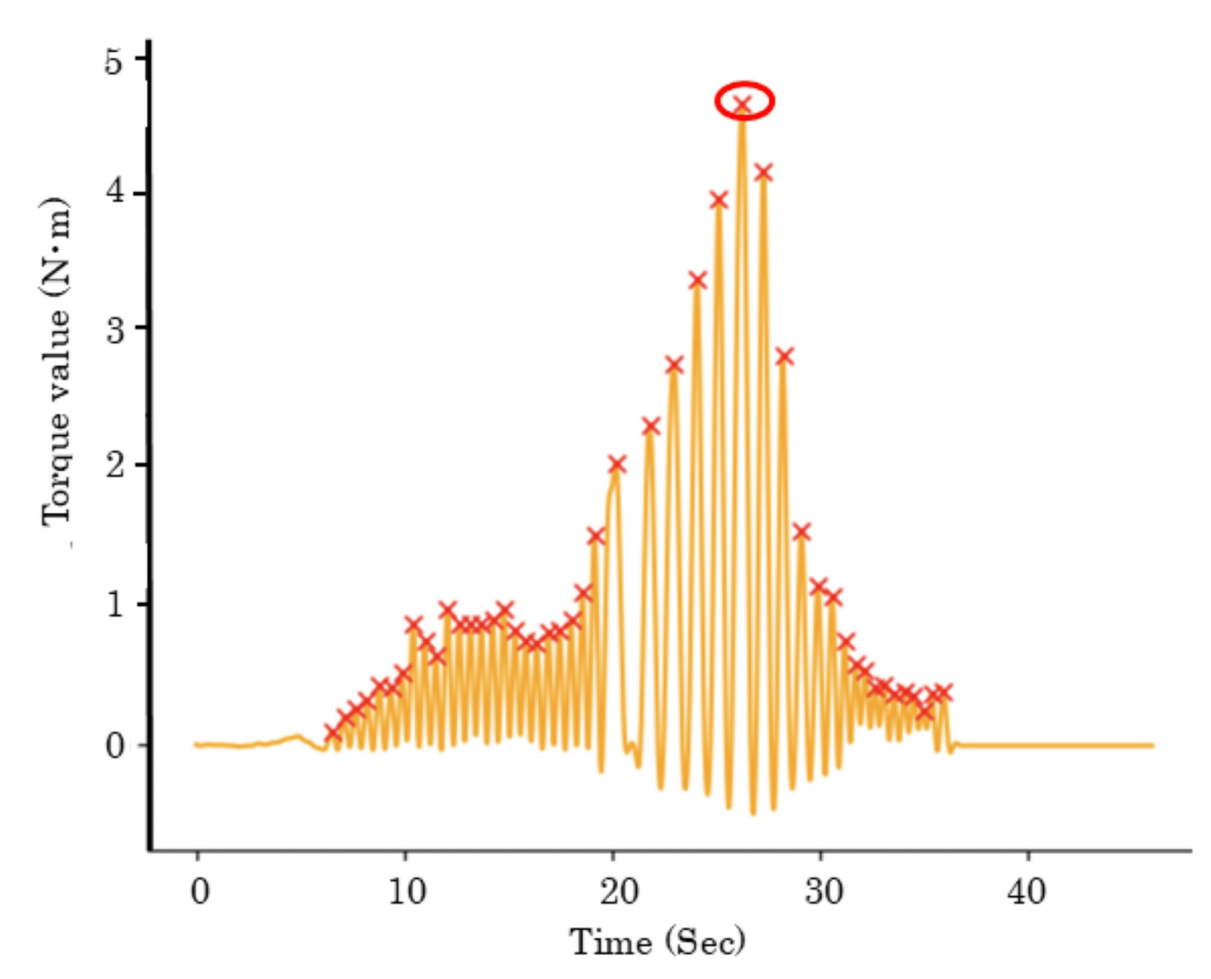
Representative waveform The representative waveform of #HA-2 sample. The waveform was smoothed using scipy.signal.butter. The peak values were automatically calculated using a Python-based library, SciPy (X). The values of torque plateaued before screw contact and then increased until screw failure. The torque value in the circle shows the maximum lag screw insertion torque.

The obtained data were analyzed using NumPy, SciPy, and statsmodels.api, the application programming interface of Python (Python Software Foundation, Wilmington, NC, USA). Additionally, the data were smoothed using a scipy.signal.butter (parameter: order of the filter 2, cut-off frequency 2, sampling frequency 2000, and type of filter low pass). The maximum lag screw insertion torque (T_Max_) was calculated using the "find_peaks" function from the scipy Python package for peak extraction (parameter: height = 0.3 and distance = 500) (Figure 4). The calculated values were expressed as the mean ± standard deviation.

Sample size

To determine the sample size, we conducted the power analysis using G*Power 3.1.9.7 (University of Düsseldorf, Düsseldorf, Germany). We used the T_Max_/f-BMD ratio as pilot data. The pilot data from group HA (13.6, 7.72, 11.3, 14.8, and 13.1) and group N (6.91, 3.88, 6.71, 10.3, and 12.0) were used to calculate Cohen’s D. The analysis parameters were set as follows: test family of t-tests, a statistical test of Wilcoxon-Mann-Whitney, type of power analysis set to a priori, a Cohen’s d of 1.4, an alpha error probability (α) of 0.05, and a desired power (1-β) of 0.90. As a result, the required total sample size was determined to be 26. Therefore, we adopted a total of 27 samples (14 samples in the HA group and 13 samples in the N group) in this study.

Statistical analysis

The values were compared between the two groups using the Welch test. The correlation between f-BMD and T_Max_ was determined using Pearson’s correlation coefficients. All analyses were performed using SPSS version 23 (IBM Corporation, Armonk, NY, USA). A p-value of <0.05 was considered statistically significant.

## Results

Twenty-seven samples were analyzed in this study: 14 samples in the HA group and 13 samples in the N group. There were four males and 10 females in the HA group, and three males and 10 females in the N group. The operated side was right in 11 cases, left in three cases in the HA group, and right in eight cases and left in five cases in the N group. The mean age of the patients was 81.4 ± 6.2 years (range, 73-91 years) and 79.0 ± 7.9 years (range, 66-90 years) in the HA and N groups, respectively (p = 0.38). f-BMD was 0.49 ± 0.11 g/cm^2^ (range, 0.24-0.69 g/cm^2^) and 0.48 ± 0.092 g/cm^2^ (range, 0.22-0.60 g/cm^2^) in the HA and N groups, respectively (p = 0.90) (Table 1).

**Table 1:** Summary of patients’ demographic characteristics, f-BMD, TMax, and TMax/f-BMD ratio HA, hydroxyapatite; f-BMD, bone mineral density of the uninjured contralateral femoral neck; T_Max_, maximum lag screw insertion torque

Parameter	N group (n = 13)	HA group (n = 14)	p
Age (mean, years)	79.0 ± 7.9 (range, 66-90)	81.4 ± 6.2 (range, 73-91)	0.38
f-BMD (mean, g/cm^2^)	0.48 ± 0.092 (range, 0.22-0.60)	0.49 ± 0.11 (range, 0.24-0.69)	0.90
T_Max_ (mean, N･m)	4.3 ± 1.9 (range, 1.6-8.2)	5.6 ± 1.9 (range, 3.0-8.6)	0.78
T_Max_/f-BMD ratio (mean, N･m･cm^2^/g)	8.9 ± 3.2 (range, 3.9-14.2)	11.7 ± 3.3 (range, 7.7-18.7)	0.036

T_Max_ was 5.6 ± 1.9 N･m (range, 3.0-8.6 N･m) and 4.3 ± 1.9 N･m (range, 1.6-8.2 N･m) in the HA and N groups, respectively (p = 0.78). However, considering bone mineral density, the T_Max_/f-BMD ratio was 11.7 ± 3.3 N･m･cm^2^/g (range, 7.7-18.7 Nm･cm^2^/g) and 8.9 ± 3.2 Nm･cm^2^/g (range, 3.9-14.2 N･m･cm^2^/g) in the HA and N groups, respectively (p = 0.036) (Table 1).

Correlation coefficients between f-BMD and T_Max_ were r = 0.50 in the HA and N groups (HA group: p = 0.068; N group: p = 0.083) (Table 2).

**Table 2:** Correlation coefficient (r) between f-BMD and TMax in the HA and N groups f-BMD, bone mineral density of the uninjured contralateral femoral neck; HA, hydroxyapatite; T_Max_, maximum lag screw insertion torque

Groups	r	Lower limit	Upper limit	p
N group (n = 18)	0.50	-0.073	0.82	0.083
HA group (n = 19)	0.50	-0.040	0.82	0.068

## Discussion

In this study, we investigated the effect of HA augmentation on the lag screw torque using extracted human femoral heads under ex vivo stable conditions. No significant differences were observed in age and f-BMD between samples from the HA and N groups. Regarding mechanical testing, we first showed that maximum lag screw insertion torque (T_Max_) was not significantly different between the HA and N groups. Additionally, moderately strong correlation coefficients were obtained between f-BMD and T_Max_ in the HA and N groups [19]. This result is consistent with previous studies [8,9]. On the other hand, considering bone mineral density, the T_Max_/f-BMD ratio was significantly higher in the HA group than in the N group. Therefore, these findings revealed that HA augmentation may increase the lag screw torque in the osteoporotic femoral head in humans. To the best of our knowledge, this is the first ex vivo biomechanical study to demonstrate that HA augmentation increases the lag screw insertion torque in extracted human femoral heads.

HA is a main component of bone and possesses good biocompatibility. Regarding the relationship between HA and bone formation, Tami et al. inserted screws with HA particles into the proximal tibia metaphysis of rats and reported the pathological assessment of HA [20]. Their study demonstrated that the amount of newly formed bone was higher in the HA group than in the control group during the remodeling stage. HA particles gradually induce bone regeneration around the screws and improve the bone-implant interface and osseointegration [20]. Furthermore, HA has been reported to improve the pullout strength of dynamic hip screws using a human femoral head in an ex vivo study [21]. Therefore, since HA not only increases the insertion torque and pullout strength of the lag screw but also promotes gradual bone regeneration after insertion, it is likely that HA provides a stronger postoperative fixation strength between bone and implant.

Neobrace® is composed of HA with a porosity of 75%, an average pore diameter of 150 µm, and an average compressive strength of 12-18 MPa. The compressive strength of Neobrace® is comparable to that of cancellous bone in the proximal femur [22]. When a lag screw is inserted, Neobrace® easily crumbles around the screw and forms granules, and the granules are presumed to increase the lag screw insertion torque. Additionally, Neobrace® has a three-dimensional interconnected porous structure that allows cell migration and vascularization into the pores, resulting in bone regeneration [23]. Taking these findings into account as a whole, Neobrace® may have the advantage of promoting implant osseointegration in intertrochanteric femoral fracture treatment. In addition, a previous biomechanical study using cellular blocks has reported that Neobrace® granulates improve both pull-out strength and rotation torque in implants with lag screws that have a relatively large bone-lag screw interface area [18]. Based on the result, HA augmentation with Neobrace® may improve fixation strength at the bone-lag screw interface. On the other hand, HA granules have the risk of intra-articular influx. However, compared to bone cement, these granules are less likely to leak into the joint and interfere with bone union [18]. Additionally, HA is the main component of bone and possesses good biocompatibility. Therefore, Neobrace® may cause fewer clinical complications than bone cement.

Lag screw insertion torque while tightening the screw was generally assessed based on the surgeon’s feeling. This conventional method makes it difficult to obtain an actual value. To solve the issue, this study used extracted human femoral heads and a dedicated jig. The screw was stopped advancing at 5 mm from the tip of the femoral head with the stopper. As the femoral head was fixed, the torque increased until the screw slipped. Consequently, Neobrace® strongly improved the T_Max_/f-BMD ratio. This result may show that HA augmentation enhances the lag screw fixation through stabilizing bone/metal thread interface. 

**Figure 5:**
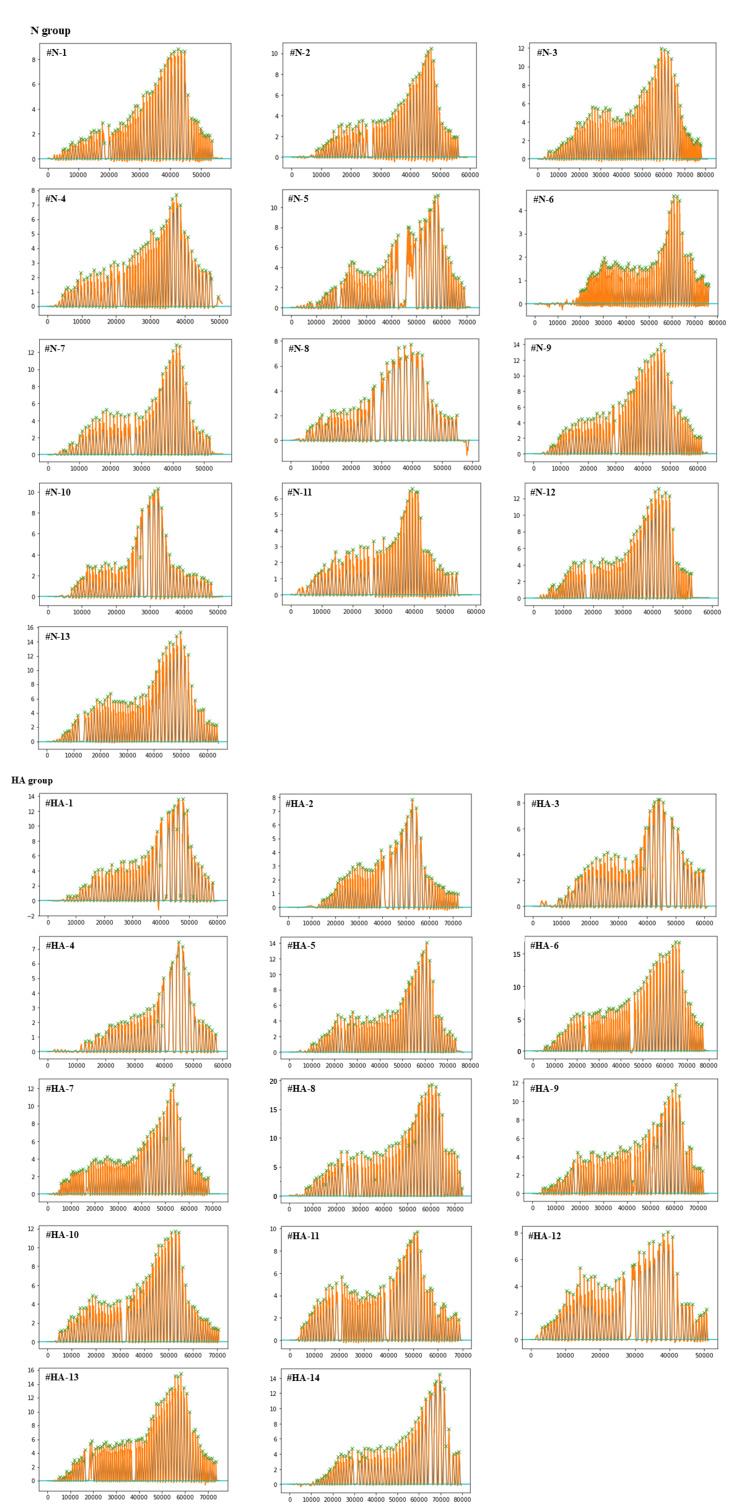
Representative waveforms for each sample These graphs show representative waveforms for each sample in both N and HA groups.

Lag screws were inserted manually in this study. While machine insertion provides more consistent results, manual insertion of lag screws can lead to variability in torque measurements despite efforts to standardize the procedure using a digital torque meter. To address this point, we have presented the recorded torque curves showing a consistent pattern across samples (Figure 5). Thus, it seems that manual insertion of lag screws may provide generally consistent results. Regarding the method of holding the actual patient femoral head with cartilage surface without slipping, the gripping section was processed with a textured surface. As a result, no slippage occurred during the experiment. Additionally, the recorded torque curves showed a consistent pattern across samples (Figure 5), indicating that the device could provide sufficient stability to fix the femoral head.

This study had some limitations. First, the sample size of patients was relatively small. As a result, although the T_Max_/f-BMD ratio showed a significant difference, other parameters may lack significance due to insufficient sample size. Second, we measured only f-BMD as the bone evaluation parameter. Third, this study uses femoral heads from elderly patients with specific characteristics undergoing bipolar hip arthroplasty. For this reason, our findings cannot be applied to a broader population, including younger or healthier individuals. Fourth, although we used a digital torque meter device to reduce variation in measurements, the speed and pressure of screw insertion may affect the torque because the screws were inserted by hand. Further investigations are needed to calculate the torque using a machine that inserts a screw at a constant speed. Also, incorporating additional metrics, such as pullout strength or fatigue testing, could provide a more comprehensive assessment of HA augmentation's efficacy.

## Conclusions

This study investigated the effect of HA material augmentation on lag screw insertion torque for femoral trochanteric fractures in an ex vivo study considering the bone density of the human femoral head. Although HA augmentation significantly improved the T_Max_/f-BMD ratio, no significant differences were observed in the T_Max_ values. This indicates that HA augmentation may improve screw fixation strength relative to bone mineral density but does not directly increase absolute torque resistance.
